# Bimetallic Metal-Organic Frameworks for Controlled Catalytic Graphitization of Nanoporous Carbons

**DOI:** 10.1038/srep30295

**Published:** 2016-07-29

**Authors:** Jing Tang, Rahul R. Salunkhe, Huabin Zhang, Victor Malgras, Tansir Ahamad, Saad M. Alshehri, Naoya Kobayashi, Satoshi Tominaka, Yusuke Ide, Jung Ho Kim, Yusuke Yamauchi

**Affiliations:** 1 Mesoscale Materials Chemistry Laboratory, International Center for Materials Nanoarchitectonics (MANA), National Institute for Materials Science (NIMS), 1-1 Namiki, Tsukuba, Ibaraki 305-0044, Japan; 2 Faculty of Science and Engineering, Waseda University, 3-4-1 Okubo, Shinjuku, Tokyo 169-8555, Japan; 3 Department of Chemistry, College of Science, King Saud University, Riyadh 11451, Saudi Arabia; 4 TOC Capacitor, 1525 Okaya, Nagano, 394-0001, Japan; 5 Australian Institute for Innovative Materials (AIIM), University of Wollongong, North Wollongong, NSW 2500, Australia

## Abstract

Single metal-organic frameworks (MOFs), constructed from the coordination between one-fold metal ions and organic linkers, show limited functionalities when used as precursors for nanoporous carbon materials. Herein, we propose to merge the advantages of zinc and cobalt metals ions into one single MOF crystal (*i.e.*, bimetallic MOFs). The organic linkers that coordinate with cobalt ions tend to yield graphitic carbons after carbonization, unlike those bridging with zinc ions, due to the controlled catalytic graphitization by the cobalt nanoparticles. In this work, we demonstrate a feasible method to achieve nanoporous carbon materials with tailored properties, including specific surface area, pore size distribution, degree of graphitization, and content of heteroatoms. The bimetallic-MOF-derived nanoporous carbon are systematically characterized, highlighting the importance of precisely controlling the properties of the carbon materials. This can be done by finely tuning the components in the bimetallic MOF precursors, and thus designing optimal carbon materials for specific applications.

Hybrid nanoporous carbon materials with controllable pore sizes, shapes, and surface properties have attracted considerable attention for the development of next-generation high performance electronic devices. Up to now, various types of carbon materials with different dimensionality (D), such as carbon-onions (0-D)^1^, carbon nanotubes (1-D)^2^, graphene (2-D)^3^, activated carbons (3-D)^4^, and templated carbons (3-D)^5^, have been explored extensively. The advantageous properties, such as suitable pore size distribution^6^,^7^,^8^, large specific surface area^9^, high electrical conductivity^10^, and doped heteroatoms^11^, are favorable for energy conversion and storage applications. Practical improvements related to a specific property causes, however, the performance associated to other properties to decrease. Thus, the rational design and synthesis of hybrid carbon materials with controlled physical and chemical properties is still a challenge and is of great interest from the viewpoint of synthetic chemistry.

In recent years, metal-organic frameworks (MOFs) have been scrutinized as convenient precursors for preparing diverse porous-carbon-based materials^12^,^13^ or metal oxides^14^,^15^, due to their regular nano-architecture constructed from various metal ions/clusters and organic ligands. Even though great progress has been made in using MOFs as precursors, the properties of the resulting porous carbons or metal oxides are limited by using only simple MOFs. As a subfamily of MOFs, zeolitic imidazolate frameworks (ZIFs), constructed from the coordination between zinc (Zn^2+^) or cobalt ions (Co^2+^) and imidazolate-type linkers^16^, have proved to be great candidates for fabricating morphology-inherited porous carbon materials. The zinc-based ZIF (ZIF-8) or cobalt-based ZIF (ZIF-67) derived carbons exhibit many advantageous properties, along with specific limitations. In detail, nanoporous carbons derived from the typical single-metal ZIFs composed of zinc ions (*e.g.*, ZIF-8) usually possess a microporous structure, large specific surface area, and high degree of nitrogen doping, but also a low degree of graphitization^17^. On the other hand, nanoporous carbons derived from the single-metal ZIFs composed of cobalt ions (*e.g.*, ZIF-67) generally possess a mesoporous structure and a high degree of graphitization, but a low specific surface area and nitrogen content^18^. These examples suggest that having only a single type of metal ions in the ZIFs comes with both advantages and disadvantages. In contrast to zinc, cobalt is able to catalytically promote the graphitization of amorphous carbon at high temperature, but at the expense of decreasing the surface area^19^ and concentration of doped heteroatoms^20^,^21^. Therefore, it is desirable to combine the advantages of zinc and cobalt ions in one single crystal (bimetallic ZIFs) in order to achieve porous carbon materials with tailored functionalities.

According to our previous research, ZIF-8 (Zn(MeIm)_2_, MeIm = 2-methylimidazolate) and ZIF-67 (Co(MeIm)_2_) are highly compatible due to their isoreticular structure and similar lattice parameters^16^,^22^. As a result, our group successfully synthesized core-shell ZIFs (ZIF-8 @ ZIF-67) by using ZIF-8 as seeds and further coating with ZIF-67 via epitaxial growth^23^. The fabrication of hetero-bimetallic ZIFs was also recently achieved via the co-precipitation of zinc and cobalt ions with MeIm^24^,^25^. Unlike the single-metal ZIFs, which only contain zinc or cobalt ions, here the zinc and cobalt ions coexist indiscriminately in the bimetallic ZIFs. As mentioned above, the zinc and cobalt ions exhibit different functionalities during carbonization. The MeIm coordinated with zinc ions can be converted into nitrogen doped carbons, and the micropores formed between the MeIm and zinc ions can be mostly retained. In contrast, the MeIm coordinated with cobalt ions tends to yield graphitic carbon, while sacrificing the microporosity and doped nitrogen. Considering this background, in the present work, we study the synthesis of nanoporous carbons using bimetallic ZIFs as precursor. The properties of the bimetallic-ZIF-derived carbons, including the specific surface area, porosity, degree of graphitization, and nitrogen doping, are precisely controlled by finely tuning the composition of the bimetallic ZIF precursors.

## Results and Discussion

**Figure 1:**
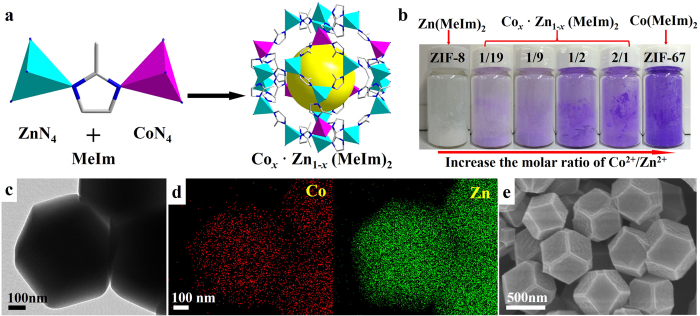
(**a**) Schematic illustration of the crystal structure of the bimetallic ZIFs (Co_*x*_·Zn_1−*x*_(MeIm)_2_). (**b**) Photograph of ZIF-8, ZIF-67, and the bimetallic ZIF (Co_*x*_·Zn_1−*x*_(MeIm)_2_) crystals. The initial molar ratios of Co^2+^/Zn^2+^ for the synthesis of each bimetallic ZIF is shown on top of the bottles in the form of an irreducible fraction. (**c**) TEM image, (**d**) elemental mapping, and (**e**) SEM image of the Co_0.1_·Zn_0.9_(MeIm)_2_.

A series of bimetallic ZIFs were prepared by reacting Co^2+^and Zn^2+^ ions with 2-methylimidazolate (MeIm) in methanolic solution. The proposed crystal structure of bimetallic ZIFs is shown in Fig. 1a, which is formed by the mixed-coordination of MeIm with Zn^2+^ and Co^2+^, respectively, based on the nets of ZnN_4_^16^ or CoN_4_^22^ tetrahedra. The bimetallic ZIF crystals are denoted as Co_*x*_·Zn_1−*x*_(MeIm)_2_, where *x*/1−*x* represent the corresponding initial molar ratio of Co^2+^/Zn^2+^ used for the synthesis, as listed in Table 1. It should be noted that ZIF-8 crystals, only consisting of zinc ions, are white. When the zinc ions are replaced by cobalt ions, the color of the obtained bimetallic ZIFs gradually changes from white to pink, lavender, and ultimately to purple (ZIF-67), as illustrated in Fig. 1b. The metal content in each bimetallic ZIF sample was precisely determined by inductively coupled plasma (ICP) analysis. As summarized in Table 1, the actual molar ratio of Co^2+^/Zn^2+^ is slightly less than the feeding molar ratio used for the synthesis, implying that the coordination interaction between zinc and MeIm is stronger than that between cobalt and MeIm. The successful preparation of bimetallic ZIFs by incorporation of Zn^2+^ and Co^2+^ into one crystal are directly demonstrated by transmission electron microscopy (TEM) and element mapping. As shown in Fig. 1c,d, the zinc and cobalt species coexist and are dispersed uniformly throughout the bimetallic Co_0.1_·Zn_0.9_(MeIm)_2_ crystals. The adjustable molar ratio of Co^2+^/Zn^2+^ in the other bimetallic ZIFs samples is also confirmed by elemental mapping (Figure S1). As discussed above, ZIF-8 and ZIF-67 are compatible thus, the resulting bimetallic ZIFs inherit the topology from both parent structures. According to the powder X-ray diffraction (XRD) patterns (Figure S2), the diffraction peaks of the bimetallic ZIFs match well with the single-metal ZIF-8 and ZIF-67. The absence of shifted peaks reflects the crystal compatibility between the parent structures as the lattice seems not to suffer from any distortions. The SEM images in Fig. 1e and Figure S3b–e show that the series of bimetallic ZIFs have a rhombic dodecahedral shape identical to single-metal ZIF-8 and ZIF-67 (Figure S3a,f).

**Figure 2:**
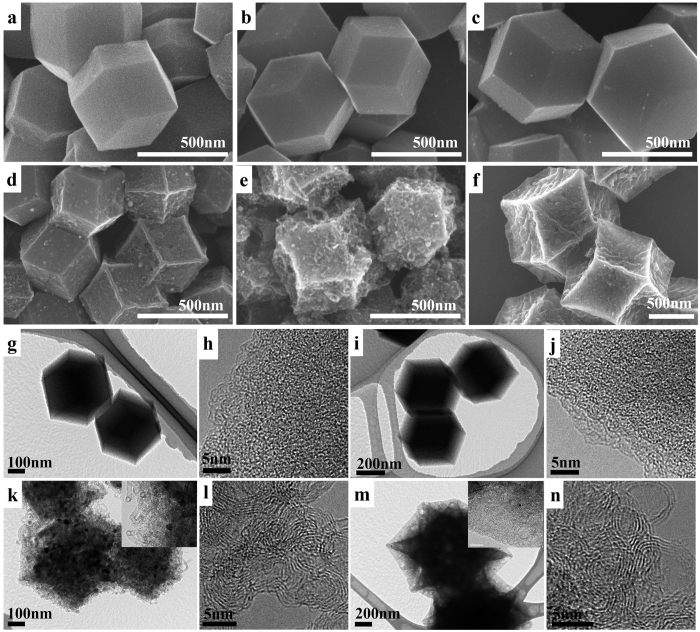
SEM images of the (**a**) C-ZIF-8, (**b**) C-1/19, (**c**) C-1/9, (**d**) C-1/2, (**e**) C-2/1 and (**f**) C-ZIF-67. TEM and high-resolution TEM images of the representative samples (**g,h**) C-ZIF-8, (**i,j**) C-1/9, (**k,l**) C-2/1 and (**m,n**) C-ZIF-67. The insets in (**k**,**m**) show higher magnification images of the edges.

In order to investigate the effect of the Co^2+^/Zn^2+^ molar ratio on the degree of graphitization, specific surface area, and pore size distribution of the bimetallic-ZIF-derived carbon, the series of bimetallic ZIFs were carbonized at an elevated temperature. As shown in the thermogravimetric (TG) curves (Figure S4), the thermal stability of bimetallic ZIF crystals under a N_2_ atmosphere gradually becomes lower along with the increased Co^2+^/Zn^2+^ ratios, which corresponds to the decreased thermal stability from single-metal ZIF-8 to ZIF-67. The weight of ZIF-8, ZIF-67, and bimetallic ZIF (Co_*x*_·Zn_1−*x*_(MeIm)_2_) crystals decreases rapidly as the temperature increases, ultimately yielding to ~50 wt% at 900 °C. During heat treatment under inert atmospheres, most the organic linkers thermally converted into the carbon matrix, while some parts also decomposed and evaporated as small molecules. The porous carbon materials derived from the bimetallic ZIFs (Co_*x*_·Zn_1−*x*_(MeIm)_2_) are labelled as C-*y* (*y* = *x*/1−*x*). C-ZIF-8 and C-ZIF-67 are also prepared for comparison by respectively using single-metal ZIF-8 and ZIF-67 as the precursors. As shown in the SEM images (Fig. 2a–f), all of the carbon materials kept the rhombic dodecahedral morphology inherited from the parent ZIFs. It is worth mentioning that the surfaces of C-ZIF-8, C-1/19, and C-1/9 are smooth (Fig. 2a–c). When the molar ratio of Co^2+^/Zn^2+^ increases above 1/2, however, the derived carbon materials C-1/2, C-2/1, and C-ZIF-67 are found to have a rough surface and shrunken facets (Fig. 2d–f). A detailed characterization by TEM and high-resolution TEM reveals that the smooth samples of C-ZIF-8 and C-1/9 only consist of amorphous carbon (Fig. 2g–j) whereas the rough samples of C-2/1 and C-ZIF-67 are composed of graphitic carbon sheets (Fig. 2k–n). These results suggest that the carbonized bimetallic ZIFs can be effectively graphitized in the presence of enough cobalt species, which explains the rough surface and distorted facets.

During carbonization, the MeIm from the bimetallic ZIF is converted into a carbon state and the coexisting Zn^2+^and Co^2+^ ions are thermally reduced to metallic Zn and Co nanoparticles, respectively. Incorporating catalytic active transition metals into the carbon precursor (*e.g.* Fe, Ni, Co) has been demonstrated as an effective approach for catalytic graphitization of amorphous carbon via solid-state transformation process^26^,^27^. As a result, the MeIm organic linkers that surround the cobalt ions tend to be catalytically converted into graphitic carbon. However, the organic linkers that surround zinc ions tend to yield amorphous carbon because a part of the zinc evaporates during the high temperature treatment and the residual zinc nanoparticles have a weak catalytic graphitization effect^25^. In other words, the degree of graphitization of C-*y* can be easily controlled by adjusting the molar ratio of Co^2+^/Zn^2+^ in the parent bimetallic ZIFs.

A close observation of the representative sample C-2/1 by high magnification SEM and TEM images (Figure S5) confirms the presence of thin graphitic carbon nanotubes grown on the surfaces of C-2/1. Although C-1/2 and C-ZIF-67 also consist of graphitic carbon, the presence of carbon nanotubes could not be observed (Fig. 2d,f). This suggests that there is an optimal ratio of Co^2+^/Zn^2+^ in the bimetallic ZIFs to favor the growth of carbon nanotubes under inert atmosphere only. In this case, the zinc species in the bimetallic ZIFs separates from the cobalt species and prevents the excessive growth of cobalt nanoparticles during carbothermal reduction of cobalt ions, resulting in the formation of abundant, dispersed, and catalytically active cobalt nanoparticles. At the same time, these cobalt nanoparticles are surrounded by a suitable amount of carbon atoms that will be effectively catalytically converted to be carbon nanotubes^28^.

**Figure 3:**
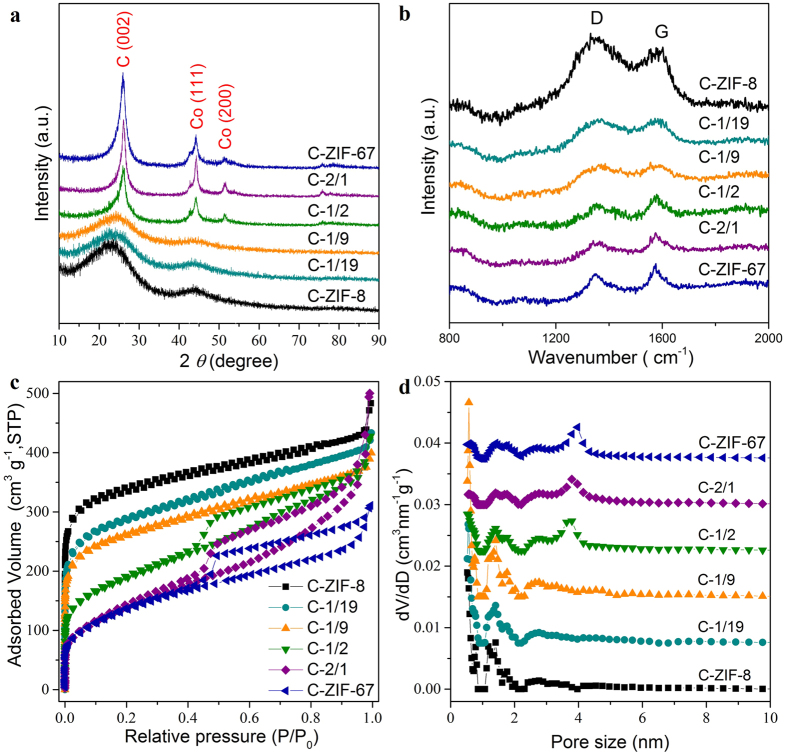
(**a**) Wide-angle PXRD patterns, (**b**) Raman spectra, (**c**) N_2_ adsorption-desorption isotherms, (**d**) pore-size distributions, as estimated by the DFT method, of the C-ZIF-8, C-*y*, and C-ZIF-67 samples. For clarity, the isotherms for C-ZIF-8 are offset by 40 cm^3^ · g^−1^. The pore-size distribution curves for C-1/19, C-1/9, C-1/2, C-2/1, and C-ZIF-67 are offset vertically by 0.0075, 0.015, 0.0225, 0.03, and 0.0375 cm^3^ · nm^−1^ · g^−1^, respectively.

The degree of graphitization of carbon materials can be characterized by XRD and Raman spectra. As shown in Fig. 3a, the C-ZIF-8, C-1/19, and C-1/9 samples only exhibit two broad diffraction peaks at 23° and 44°, which are indexed to the (002) and (101) diffraction planes of amorphous carbon^23^. The broad diffraction peak at around 23° shift slightly toward higher angles as the cobalt content is increased from C-ZIF-8 to C-1/19, and to C-1/9, indicating the gradual formation of graphitized carbon. In the case of C-1/2, C-2/1, and C-ZIF-67, which have higher ratios of cobalt, the (002) diffraction peak is observed at 26°, indicating highly graphitic carbon states^28^. These results confirm the importance of cobalt ions on the degree of graphitization in the bimetallic-ZIF-derived carbon. In addition, as revealed by the XRD patterns in Figure S6, the elevated calcination temperature from 800 to 900 °C also is quite critical to promote the graphitization of carbon, thus helps to magnify the distinction of graphitization degree in the series of bimetallic-ZIF-derived carbons in this study. The increased degree of graphitization from C-ZIF-8, to C-1/19, C-1/9, C-1/2, C-2/1, and C-ZIF-67 was further observed by Raman spectroscopy (Fig. 3b). Each carbon sample displays two vibration bands. The D band located at 1360 cm^−1^ corresponds to the vibrations of disordered carbon or defects, while the G band located at 1590 cm^−1^ is related to the vibrations of *sp*^2^-bonded graphitic carbon sheets^29^. The intensity ratio between the D and G band (I_D_/I_G_) provides a good insight on the degree of graphitization for comparative studies. As shown in Table 2, the value of I_D_/I_G_ for C-ZIF-8, C-*y*, and C-ZIF-67 decreases with increasing the cobalt content in the bimetallic ZIF precursors, suggesting an improved graphitization.

In addition to the diffraction peaks of carbon, C-1/2, C-2/1, and C-ZIF-67 display other intense diffraction peaks at 44° and 51°, which can be attributed to the (111) and (200) diffractions of face-centered-cubic metallic Co nanoparticles (Fig. 3a). The average size of the Co nanoparticles is estimated to be 9.2, 11.8, and 11.9 nm for C-1/2, C-2/1, and C-ZIF-67, respectively, by using the Scherrer equation^30^. Although most of the cobalt nanoparticles in the carbon matrix can be removed via acid etching to form the pores, a small fraction of Co nanoparticles wrapped in circular graphitic carbon layers which formed during the catalytic graphitization, are protected from acid erosion and kept embedded in the carbon matrix^31^,^32^. The spatial distribution of carbon, nitrogen, and cobalt was detected by elemental mapping (Figure S7). Zinc completely disappeared from each carbon sample, and cobalt was also rarely observed. Even when the ZIFs containing high content of Co ions are used as the precursor, the resulting cobalt content is only 2.15 wt% and 1.90 wt% in C-2/1 and C-ZIF-67, respectively. From CHN analysis, it is found that the level of doped nitrogen (relative weight ratio of nitrogen to carbon) is 24.0, 23.4, 23.0, 6.2, 3.5, and 2.7%, for C-ZIF-8, C-1/19, C-1/9, C-1/2, C-2/1, and C-ZIF-67, respectively. This confirms that the nitrogen heteroatoms, originating from the MeIm organic linker, are preserved in the carbon matrix after the high temperature treatment. However, the content of nitrogen decreases along with the increased cobalt ratios in the bimetallic ZIF precursors which is probably due to the breakdown of C-N bond during the catalytically graphitization promoted by cobalt nanoparticles.

The porosity of the carbon samples was investigated by N_2_ adsorption-desorption isotherms. The sharp nitrogen uptake at low relative pressure stage (*P*/*P*_0_ < 0.05) is generally attributed to the strong nitrogen adsorption into micropores. As shown in Fig. 3c, the C-ZIF-8, C-1/19, and C-1/9 samples exhibit a much higher nitrogen uptake at low relative pressure compared with the C-1/2, C-2/1, and C-ZIF-67 samples, indicating higher microporosity. All of the samples have gradual nitrogen uptakes in the middle relative pressure range from 0.3 to 0.9, implying the presence of mesopores with wide size distributions. Unlike C-ZIF-8, C-1/19, and C-1/9, the C-1/2, C-2/1, and C-ZIF-67 samples display a clear hysteresis loop, which is generally due to presence of abundant, random, bumpy, and non-uniform mesopores^33^. The pore size distributions (Fig. 3d), calculated by the density functional theory (DFT) method, further prove that the pores in C-*y* gradually move from micropores towards mesopores with increasing the cobalt content in the bimetallic ZIF precursors.

The specific surface areas of C-*y* are also closely related to the cobalt ratios in the bimetallic ZIFs. As summarized in Table 2, the surface areas decrease from C-ZIF-8 (925 m^2^ · g^−1^), to C-1/19 (890 m^2^ · g^−1^), C-1/9 (781 m^2^ · g^−1^), C-1/2 (643 m^2^ · g^−1^), C-2/1 (502 m^2^ · g^−1^), and finally to C-ZIF-67 (450 m^2^ · g^−1^). The surface area of C-ZIF-8 (925 m^2^ · g^−1^, carbonized at 900 °C) is lower than that of our previously reported ZIF-8-derived-carbon (1499 m^2^ · g^−1^, carbonized at 800 °C)^23^, probably due to the collapse of some nanopores during the calcination at higher temperature. In addition, the ratio of the microporous surface area to the total surface area gradually decreases as the Co^2+^/Zn^2+^ molar ratio increases (Table 2). These results suggest that the microporosity in the carbons is inherited from the microporous ZIF precursor and that it is sacrificed during the graphitization of amorphous carbon catalyzed by the cobalt nanoparticles. Meanwhile, the mesopores are generated by the carbonization process and the subsequent removal of metal nanoparticles. Thus, hierarchically crosslinked micro/mesoporous structures are developed, and are expected to promote electrolyte penetration and to lower the diffusion resistance when used as electrode materials in electrochemical devices.

The electric state of N in the carbon was investigated by X-ray photoelectron spectroscopy (XPS). As shown in Figure S8, the high resolution spectrum of the N 1s peak of the representative C-1/19 sample can be mainly fitted with two peaks centered at ~398.6 and ~400.4 eV, which are assignable to pyridinic-N and graphitic-N, respectively^34^. This result demonstrates that the N atoms in the original pentagonal ring of the imidazole units are mainly doped into the carbon framework through two distinct mechanisms following the carbonization process. Pyridinic N, referring to the sp^2^-hybridized N atoms bonded with two sp^2^-hybridized C neighbours via σ-bonds, possesses one lone-pair of electrons in the graphene plane, and contributes one electron to the conjugated π system^35^. In the graphitic N configuration, three sp^2^-hybridized N valence electrons form three σ-bonds with three sp^2^-hybridized C neighbours, one electron fills the π-orbitals, and the fifth electron enters the π^*^-states of the conduction band^36^. According to another report, the fifth electron is distributed in the local network of the carbon π-system whereas a part of the charge localizes on the graphitic N dopant and electronically couples to its nearest C neighbours^37^.

**Figure 4:**
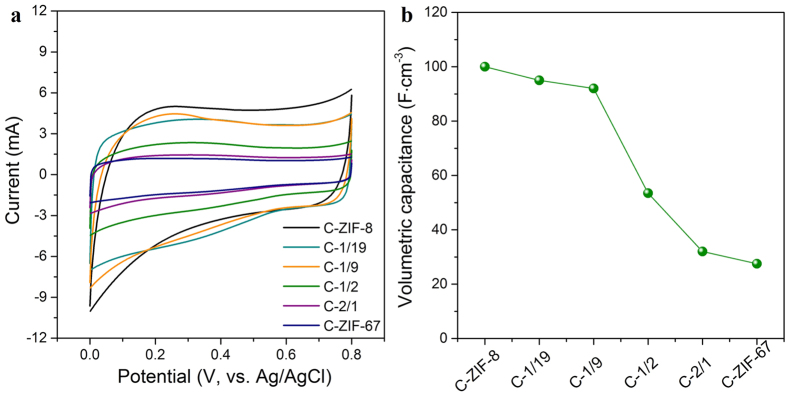
(**a**) CV curves and (**b**) volumetric capacitance for C-ZIF-8, C-*y*, and C-ZIF-67 samples at a scan rate of 20 mV · s^−1^.

To the best of our knowledge, this is the first example of a facile control over the degree of graphitization, pore size distribution, and nitrogen doping in carbons by utilizing bimetallic MOFs with adjustable compositions. Furthermore, the series of bimetallic-MOF-derived nanoporous carbons doped with heteroatoms, which features high surface area and a continuous conductive framework, have been investigated as promising compounds for electrodes in supercapacitor application using a standard three-electrode system. Cyclic voltammetry (CV) studies were carried out in a potential window ranging from 0.0 to 0.8 V. The CV curves for the samples show a quasi-rectangular shape (Fig. 4a). The volumetric performance is an important technological metric for energy storage devices to meet realistic application. The variation of the volumetric capacitance values obtained for different samples is shown in Fig. 4b. The volumetric capacitances are calculated to be 100, 95, 93, 54, 33, and 29 F∙cm^−3^ for C-ZIF-8, C-1/19, C-1/9, C-1/2, C-2/1, and C-ZIF-67, respectively, at a scan rate of 20 mV · s^−1^. From these values, it can be clearly found that the capacitance decreases as the Co^2+^/Zn^2+^ ratio in the bimetallic ZIF precursor is increased. These results are consistent with the N_2_ adsorption-desorption data, which indicates that the surface area as well as the number of micropores decrease as the cobalt content increases (Table 2). The capacitance retention at a high scan speed is one of the most important parameters for high performance supercapacitors. As shown in Figure S9, the capacitance retention for the C-ZIF-8 sample is found to be only 38% after cycling at a 200 mV∙s^−1^, which is significantly lower than 71%, 85%, and 84% for the C-1/19, C-1/2, and C-ZIF-67 samples, respectively. The increased retention values can be explained by (i) the decreased concentration of micropores and increased concentration of mesopores which allow fast intercalation/de-intercalation of ions in the material, and (ii) the higher conductivity of the carbon matrix, owing to the increased degree of carbon graphitization^38^,^39^. Among all the samples, C-1/19 shows both a high volumetric capacitance and capacitance retention. By using C-1/19 sample as both positive and negative electrodes, a symmetric supercapacitor cell was fabricated and the details are shown in Figure S10.

## Conclusion

In summary, bimetallic ZIFs (Co_*x*_·Zn_1−*x*_(MeIm)_2_) have been successfully prepared due to the crystal compatibility between ZIF-8 (Zn(MeIm)_2_) and ZIF-67 (Co(MeIm)_2_). Unlike the single-metal ZIFs, which only contain zinc or cobalt ions, the zinc and cobalt ions coexist in the bimetallic ZIFs and support different functionalities during the carbonization process. As a result, the physical and chemical properties of the bimetallic-ZIF-derived carbon can be designed via simply and precisely adjusting the ratio of Co^2+^/Zn^2+^ in the bimetallic ZIF precursor. Therefore, this work offers a practical way to achieve optimal properties in carbon materials for specific applications by easily tailoring the components of the bimetallic ZIFs precursors.

## Methods

### Preparation of bimetallic ZIFs (Co_x_·Zn_1−x_(MeIm)_2_)

In a typical synthesis, Co(NO_3_)_2_∙6H_2_O and Zn(NO_3_)_2_∙6H_2_O were dissolved in 30 mL of methanol to form a clear solution, followed by the addition of 2-methylimidazole (984 mg, 12 mmol) dissolved in 10 mL of methanol. After thoroughly mixing by continuous stirring for 10 min, the solution was then transferred into an autoclave and was incubated at 100 °C for 12 hours. After cooling to room temperature, the resulting crystals were collected by centrifugation, washed with methanol, and dried at 60 °C. The molar ratio of Co^2+^/Zn^2+^ in the obtained bimetallic crystals was adjustable over a wide range by varying the initial metallic precursor ratio. The total molarity of Co^2+^and Zn^2+^ was fixed to be 3 mmol during the synthesis. As a result, a series of bimetallic ZIFs were prepared and categorized as Co_*x*_·Zn_1−*x*_(MeIm)_2_, where *x*/1−*x* represents the corresponding initial molar ratio of Co^2+^/Zn^2+^ and the obtained bimetallic ZIFs are denoted as Co_0.05_·Zn_0.95_(MeIm)_2_, Co_0.1_·Zn_0.9_(MeIm)_2_, Co_0.33_·Zn_0.67_(MeIm)_2_, and Co_0.67_·Zn_0.33_(MeIm)_2_, respectively. Two single-metal ZIFs, ZIF-8 (Zn(MeIm)_2_) and ZIF-67 (Co(MeIm)_2_), were also prepared by adding only Zn^2+^ or Co^2+^, respectively.

### Carbonization of bimetallic ZIFs (Co_x_·Zn_1−x_(MeIm)_2_)

Bimetallic ZIFs (Co_*x*_·Zn_1−*x*_(MeIm)_2_), ZIF-8, and ZIF-67 crystals were thermally converted into nanoporous carbon through carbonization under flowing argon at 900 °C for 3 hours with a heating rate of 2 °C·min^−1^. The Zn and Co species were removed by extensive washing with HF solution (10 wt%). The nanoporous carbons converted from bimetallic ZIFs (Co_*x*_·Zn_1*−x*_(MeIm)_2_) are denoted as C-*y* (*y* = *x*/1−*x*, in the form of an irreducible fraction). Thus, the nanoporous carbon converted from the bimetallic ZIFs Co_0.05_·Zn_0.95_(MeIm)_2_, Co_0.1_·Zn_0.9_(MeIm)_2_, Co_0.33_·Zn_0.67_(MeIm)_2_, and Co_0.67_·Zn_0.33_(MeIm)_2_ are designated as C-1/19, C-1/9, C-1/2, and C-2/1, respectively. C-ZIF-8 and C-ZIF-67 are also prepared for comparison by using ZIF-8 and ZIF-67 as the precursors, respectively.

## Additional Information

**How to cite this article**: Tang, J. *et al.* Bimetallic Metal-Organic Frameworks for Controlled Catalytic Graphitization of Nanoporous Carbons. *Sci. Rep.* **6**, 30295; doi: 10.1038/srep30295 (2016).

## Supplementary Material

Supplementary Information

## Figures and Tables

**Table 1:** Summary of the molar ratios of Co^2+^/Zn^2+^ in bimetallic ZIFs.

Sample	ICP determined molar ratio of Co^2+^/Zn^2+^	Feeding molar ratio of Co^2+^/Zn^2+^
Co_0.05_·Zn_0.95_(MeIm)_2_	0.027	0.053
Co_0.1_·Zn_0.9_(MeIm)_2_	0.065	0.111
Co_0.33_·Zn_0.67_(MeIm)_2_	0.356	0.500
Co_0.67_·Zn_0.33_(MeIm)_2_	1.886	2.000

**Table 2:** The surface areas and total pore volumes calculated from N_2_ adsorption-desorption isotherms, and the ratios of D band to G band estimated from Raman spectra are summarized.

Sample	*S*_BET_ (m^2^·g^−1^)	*S*_micro_ (m^2^·g^−1^)	*S*_micro_ /*S*_BET_	*V*_pore_ (cm^3^·g^−1^)	*V*_micro_ (cm^3^·g^−1^)	*V*_micro_ /*V*_pore_	I_D_/I_G_
C-ZIF-8	925	541	58.5%	0.57	0.29	50.9%	1.19
C-1/19	890	426	47.9%	0.58	0.23	39.7%	1.00
C-1/9	781	250	32.0%	0.52	0.14	26.9%	1.00
C-1/2	643	123	19.1%	0.54	0.06	11.1%	0.67
C-2/1	502	57	11.4%	0.53	0.03	5.7%	0.67
C-ZIF-67	450	41	9.1%	0.43	0.01	2.3%	0.67

## References

[b1] Pech D. *et al.* Ultrahigh-power micrometre-sized supercapacitors based on onion-like carbon. Nat. Nanotechnol. 5, 651–654 (2010). 20711179 10.1038/nnano.2010.162

[b2] An K. H. *et al.* Electrochemical properties of high-power supercapacitors using single-walled carbon nanotube electrodes. Adv. Funct. Mater. 11, 387–392 (2001).

[b3] Wang X. *et al.* Three-dimensional strutted graphene grown by substrate-free sugar blowing for high-power-density supercapacitors. Nat. Commun. 4, 2905 (2013). 24336225 10.1038/ncomms3905 PMC3905699

[b4] Hulicova-Jurcakova D., Seredych M., Lu G. Q. & Bandosz T. J. Combined effect of nitrogen- and oxygen-containing functional groups of microporous activated carbon on its electrochemical performance in supercapacitors. Adv. Funct. Mater. 19, 438–447 (2009).

[b5] Qiao Z.-A. *et al.* Controlled synthesis of mesoporous carbon nanostructures via a “silica-assisted” strategy. Nano Lett. 13, 207–212 (2013). 23256449 10.1021/nl303889h

[b6] Wang D.-W., Li F., Liu M., Lu G. Q. & Cheng H.-M. 3D aperiodic hierarchical porous graphitic carbon material for high-rate electrochemical capacitive energy storage. Angew. Chem., Int. Ed. 47, 373–376 (2008). 10.1002/anie.200702721 18022983

[b7] Chmiola J. *et al.* Anomalous increase in carbon capacitance at pore sizes less than 1 nanometer. Science 313, 1760–1763 (2006). 16917025 10.1126/science.1132195

[b8] Zhang L. *et al.* Controlling the effective surface area and pore size distribution of sp^2^ carbon materials and their impact on the capacitance performance of these materials. J. Am. Chem. Soc. 135, 5921–5929 (2013). 23565654 10.1021/ja402552h

[b9] Aricò A. S., Bruce P., Scrosati B., Tarascon J. M. & Schalkwijk W. V. Nanostructured materials for advanced energy conversion and storage devices. Nat. Mater. 4, 366–377 (2005). 15867920 10.1038/nmat1368

[b10] Hu J. *et al.* Preparation of highly graphitized porous carbon from resins treated with cr^6+^ -containing wastewater for supercapacitors. J. Mater. Chem. A 1, 6558–6562 (2013).

[b11] Hulicova-Jurcakova D. *et al.* Highly stable performance of supercapacitors from phosphorus-enriched carbons. J. Am. Chem. Soc. 131, 5026–5027 (2009). 19317481 10.1021/ja809265m

[b12] Liu B., Shioyama H., Akita T. & Xu Q. Metal-Organic framework as a template for porous carbon synthesis. J. Am. Chem. Soc. 130, 5390–5391 (2008). 18376833 10.1021/ja7106146

[b13] Jiang H.-L. *et al.* From metal–organic framework to nanoporous carbon: toward a very high surface area and hydrogen uptake. J. Am. Chem. Soc. 133, 11854–11857 (2011). 21751788 10.1021/ja203184k

[b14] Cao X. *et al.* Metal oxide-coated three-dimensional graphene prepared by the use of metal–organic frameworks as precursors. Angew. Chem., Int. Ed. 53, 1404–1409 (2014). 10.1002/anie.201308013 24459058

[b15] Salunkhe R. R. *et al.* Asymmetric supercapacitors using 3d nanoporous carbon and cobalt oxide electrodes synthesized from a single metal–organic framework. ACS Nano 9, 6288–6286 (2015). 25978143 10.1021/acsnano.5b01790

[b16] Park K. S. *et al.* Exceptional chemical and thermal stability of zeolitic imidazolate frameworks. Proc. Natl. Acad. Sci. USA 103, 10186–10191 (2006). 16798880 10.1073/pnas.0602439103 PMC1502432

[b17] Torad N. L. *et al.* Facile synthesis of nanoporous carbons with controlled particle sizes by direct carbonization of monodispersed ZIF-8 crystals. Chem. Commun. 49, 2521–2523 (2013). 10.1039/c3cc38955c 23423451

[b18] Torad N. L. *et al.* Direct synthesis of MOF-derived nanoporous carbon with magnetic Co nanoparticles toward efficient water treatment. Small 10, 2096–2107 (2014). 24610684 10.1002/smll.201302910

[b19] Chmiola J., Yushin G., Dash R. & Gogotsi Y. Effect of pore size and surface area of carbide derived carbons on specific capacitance. J. Power Sources 158, 765–772 (2006).

[b20] Li X.-H., Kurasch S., Kaiser U. & Antonietti M. Synthesis of monolayer-patched graphene from glucose. Angew. Chem., Int. Ed. 51, 9689–9692 (2012). 10.1002/anie.201203207 22907631

[b21] Hulicova D., Yamashita J., Soneda Y., Hatori H. & Kodama M. Supercapacitors prepared from melamine-based carbon. Chem. Mater. 17, 1241–1247 (2005).

[b22] Banerjee R. *et al.* High-throughput synthesis of zeolitic imidazolate frameworks and application to CO_2_ capture. Science 319, 939–943 (2008). 18276887 10.1126/science.1152516

[b23] Tang J. *et al.* Thermal conversion of core−shell metal−organic frameworks: a new method for selectively functionalized nanoporous hybrid carbon. J. Am. Chem. Soc. 137, 1572–1580 (2015). 25580699 10.1021/ja511539a

[b24] Wu R. *et al.* Porous spinel Zn_*x*_Co_3−*x*_O_4_ hollow polyhedra templated for high-rate lithium-ion batteries. ACS Nano 8, 6297–6303 (2014). 24833068 10.1021/nn501783n

[b25] Chen Y.-Z. *et al.* From bimetallic metal-organic framework to porous carbon: high surface area and multicomponent active dopants for excellent electrocatalysis. Adv. Mater. 27, 5010–5016 (2015). 26193083 10.1002/adma.201502315

[b26] Loh G. C. & Baillargeat D. Graphitization of amorphous carbon and its transformation pathways. J. Appl. Phys. 114, 033534 (2013).

[b27] Su P. *et al.* Nitrogen-doped carbon nanotubes derived from Zn-Fe-ZIF nanospheres and their application as efficient oxygen reduction electrocatalysts with *in situ* generated iron species. Chem. Sci. 4, 2941–2946 (2013).

[b28] Xia B. Y. *et al.* A metal–organic framework-derived bifunctional oxygen electrocatalyst. Nat. Energy 1, 15006 (2016).

[b29] Zhang R., Liu Y., Yu L., Li Z. & Sun S. Preparation of high-quality biocompatible carbon dots by extraction, with new thoughts on the luminescence mechanisms. Nanotechnology 24, 225601 (2013). 23644814 10.1088/0957-4484/24/22/225601

[b30] Tang J. *et al.* Synthesis and electrochemical characterization of N-doped partially graphitized ordered mesoporous carbon-Co composite. J. Phys. Chem. C 117, 16896–16906 (2013).

[b31] Lu A.-H. *et al.* Highly stable carbon-protected cobalt nanoparticles and graphite shells. Chem. Commun. 98–100 (2005). 10.1039/b414146f 15614385

[b32] Tang J. *et al.* Cage-type highly graphitic porous carbon-Co_3_O_4_ polyhedron as the cathode of lithium−oxygen batteries. ACS Appl. Mater. Interfaces 8, 2796–2804 (2016). 26788868 10.1021/acsami.5b11252

[b33] Sing K. S. W. *et al.* Reporting physisorption data for gas/solid systems with special reference to the determination of surface area and porosity. Pure Appl. Chem. 57, 603–619 (1985).

[b34] Zheng F., Yang Y. & Chen Q. High lithium anodic performance of highly nitrogen-doped porous carbon prepared from a metal-organic framework. Nat. Commun. 5, 5261 (2014). 25374050 10.1038/ncomms6261

[b35] Lai L. *et al.* Exploration of the active center structure of nitrogen-doped graphene-based catalysts for oxygen reduction reaction. Energy Environ. Sci. 5, 7936–7942 (2012).

[b36] Usachov D. *et al.* Nitrogen-doped graphene: efficient growth, structure, and electronic properties. Nano Lett. 11, 5401–5407 (2011). 22077830 10.1021/nl2031037

[b37] Schiros T. Connecting dopant bond type with electronic structure in N-doped graphene. Nano Lett. 12, 4025–4031 (2012). 22746249 10.1021/nl301409h

[b38] Liu B., Shioyama H., Jiang H., Zhang X. & Xu Q. Metal–organic framework (MOF) as a template for syntheses of nanoporous carbons as electrode materials for supercapacitor. Carbon. 48, 456–463 (2010).

[b39] Zhu H., Yin J., Wang X., Wang H. & Yang X. Microorganism-derived heteroatom-doped carbon materials for oxygen reduction and supercapacitors. Adv. Funct. Mater. 23, 1305–1312 (2013).

