# Supplementary material for: Bimetallic Metal-Organic Frameworks for Controlled Catalytic Graphitization of Nanoporous Carbons
Source: Sci Rep. 2016 Jul 29;6:30295. doi: 10.1038/srep30295 (PMC4965863; doi:10.1038/srep30295)
Supplement: Supplementary Information [file srep30295-s1.docx]

**Bimetallic Metal**-**Organic Frameworks for Controlled Catalytic Graphitization of Nanoporous Carbons**

Jing Tang,^a,b^ Rahul R. Salunkhe,^a^ Huabin Zhang,^a^ Victor Malgras,^a^ Tansir Ahamad,^c^ Saad M. Alshehri,^c^ Naoya Kobayashi,^d^ Satoshi Tominaka,^a^ Yusuke Ide,^a^ Jung Ho Kim,*^e^ and Yusuke Yamauchi*^a,b^^,e^

*[a] Mesoscale Materials Chemistry Laboratory, International Center for Materials Nanoarchitectonics (MANA), National Institute for Materials Science (NIMS), 1-1 Namiki,* *Tsukuba, Ibaraki 305-0044, Japan.*

*[b] Faculty of Science and Engineering, Waseda University, 3-4-1 Okubo, Shinjuku, Tokyo 169-8555, Japa**n.*

*[c] Department of Chemistry, College of Science, King Saud University, Riyadh 11451, Saudi Arabia.*

*[d] TOC Capacitor, 1525 Okaya, Nagano, 394-0001, Japan.*

*[e] Australian Institute for Innovative Materials (AIIM), University of Wollongong, North Wollongong, NSW 2500, Australia.*

**Corresponding Author****s:** [jhk@uow.edu.au](mailto:jhk@uow.edu.au); [Yamauchi.Yusuke@nims.go.jp](mailto:Yamauchi.Yusuke@nims.go.jp)

**Materials and Instrumentation**

**Materials.** Zinc nitrate hexahydrate (Zn(NO_3_)_2_·6H_2_O, 99%) and cobalt nitrate hexahydrate (Co(NO_3_)_2_·6H_2_O, 99%), 2-methylimidazole (purity 99%), methanol, and hydrofluoric acid were purchased from Nacalai Tesque Reagent Co. All the chemicals were used without further purification.

**Characterization.** The morphology of the products was investigated by a Hitachi SU-8000 field-emission scanning electron microscope (SEM) at an accelerating voltage of 5 kV. Transmission electron microscopy (TEM), high-angle annular dark-ﬁeld scanning transmission electron microscopy (HAADF STEM), and elemental mapping analysis were conducted on a JEM-2100 at voltage of 200 kV. N_2_ adsorption-desorption isotherms were measured with a Quantachrome Autosorb-iQ Automated Gas Sorption System at 77 K. The surface areas of C-*y*, C-ZIF-8, and C-ZIF-67 were calculated according to the Brunauer-Emmett-Teller (BET) model by using the adsorption branch data in the relative pressure (*P*/*P*_0_) range of 0.05-0.35. The total pore volumes and pore-size distributions were estimated from the adsorption branches of the N_2_ isotherms on the basis of the density functional theory (DFT). Wide-angle powder X-ray diffraction (PXRD) patterns were acquired on a Rigaku Rint 2000 X-ray diffractometer using monochromated Cu Kα radiation (40 kV, 40 mA) at a scanning rate of 2°·min^-1^. Raman spectra were collected on a Horiba-Jovin Yvon T64000 instrument with an excitation laser wavelength of *λ* = 514.5 nm. CHN analysis was measured by Perkin Elmer 2400 CHNO Series II System. Thermogravimetric (TG) analysis was conducted on Hitachi HT-Seiko Instrument Exter 6300 TG/DTA under N_2_ atmosphere and heated from room temperature to 900 °C at a heating rate of 5 °C·min^-1^. The X-ray photoelectron spectroscopy (XPS) spectrum was acquired by using a PHI Quantera SXM (ULVAC-PHI) instrument with an Al K*α* X-ray source. The region of high-resolution N 1s spectrum ranges from 392 to 412 eV. The binding energies were calibrated via referencing to the C 1s binding energy located at 285.0 eV. The peaks of the N 1s spectrum were fitted with a Gaussian-Lorentzian sum function and a Shirley background.

**Electrochemical measurements.** The electrochemical measurements were carried out using an electrochemical workstation (CHI 660e, CH Instruments). Firstly, the electrochemical analysis was carried out using standard three-electrode measurements. Ag/AgCl and platinum were used as the reference and the counter electrode, respectively. The electrolyte used for the measurements was 1 M H_2_SO_4_. The working electrode was prepared as follows: 1 mg of bimetallic-ZIF-derived carbon material was mixed with 0.1 mg of poly(vinylidene fluoride). After adding 200 μL of N-methyl-2-pyrrolidone, the mixture was treated with ultrasonication for 20 min. The obtained homogeneous black slurry was dropped stepwise onto a graphite substrate (1 cm^2^) and dried under an infrared lamp to form a thin film. For all the samples, the mass loading per electrode was 1 mg. The thickness of the thin film estimated by cross-section SEM image is around 25 μm, the density of the active electrode material corresponds to 0.5 g∙cm^-3^. For the symmetric supercapacitor cell (SSC) measurements, two electrodes with the same mass loading were used. The positive and negative electrodes were separated from each other by a distance of 0.3 cm, without any separators, and used for the electrochemical measurements. Thus the total mass loading for both electrodes was 2 mg. The electrochemical properties of the electrodes were investigated by cyclic voltammetry (CV) and galvanostatic charge–discharge curves (CD) measurements. The gravimetric and volumetric capacitance values were calculated using cyclic voltammetry and galvanostatic charge-discharge measurements and the following equations:

$C_{g}=\frac{1}{ms (V_{f}-V_{i})}\int_{V_{i}}^{V_{f}} I\left( V \right)dV$ (1)

$C_{g}= \frac{I \times\Delta t}{m\times\Delta V}$ (2)

$C_{v}= C_{g}\times\rho$ (3)

where *C_g_* is the gravimetric capacitance (F∙g^-1^), *C_v_* volumetric capacitance (F∙cm^-3^), *s* is the potential scan rate, *V* the is potential window, *I* is the current (A), *t* is the discharg[e](#_ENREF_1) time, *m* is the mass in grams, and *ρ* is the density of the active electrode material.

**Figure S1**


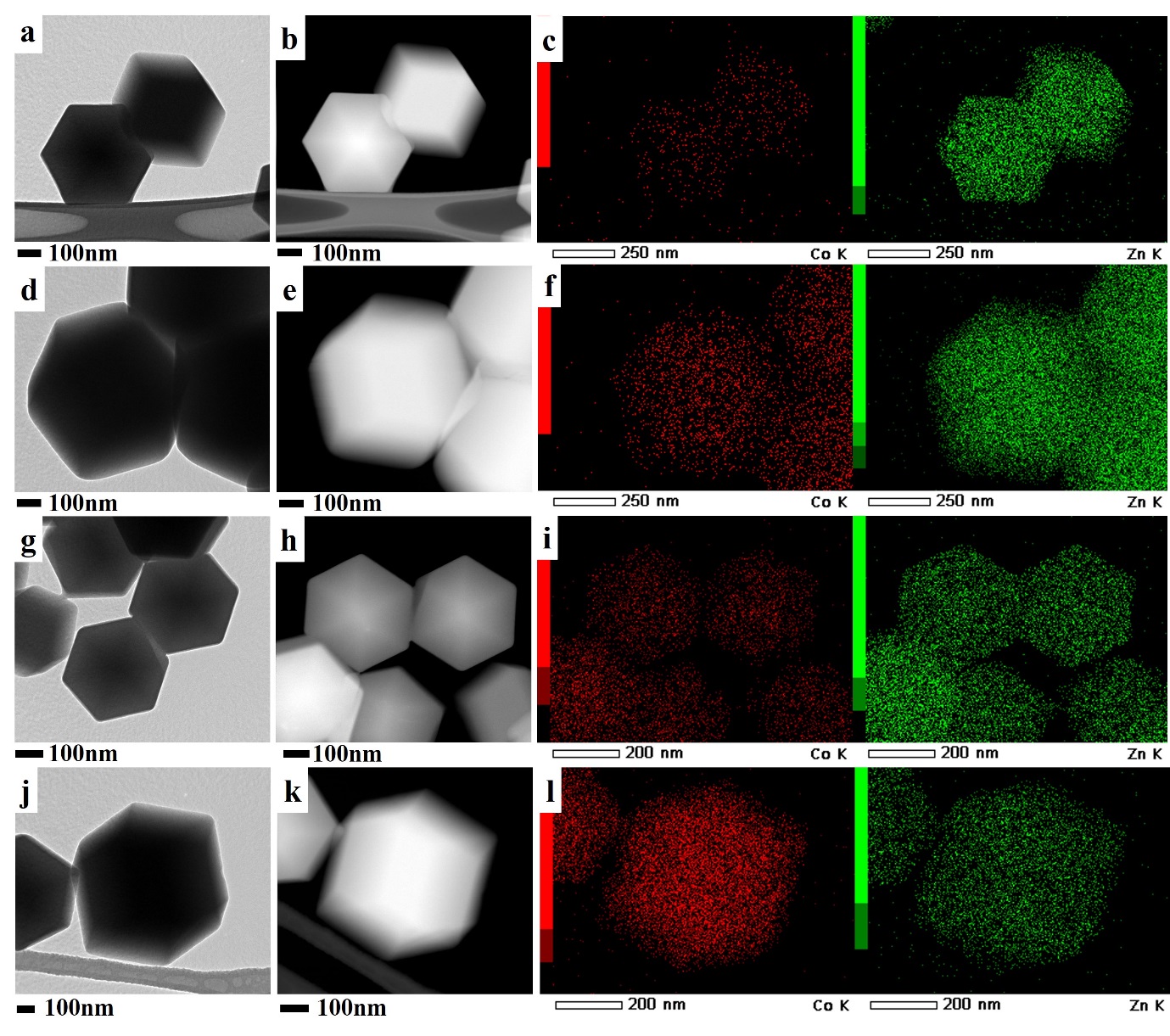


**Figure S1.** (a, d, g, j) TEM images, (b, e, h, k) HAADF STEM images, and (c, f, i, l) elemental mappings of the bimetallic ZIFs. (a-c) Co_0.05_·Zn_0.95_(MeIm)_2_, (d-f) Co_0.1_·Zn_0.9_(MeIm)_2_, (g-i) Co_0.33_·Zn_0.67_(MeIm)_2_, and (j-l) Co_0.67_·Zn_0.33_(MeIm)_2_.

**Figure S2**


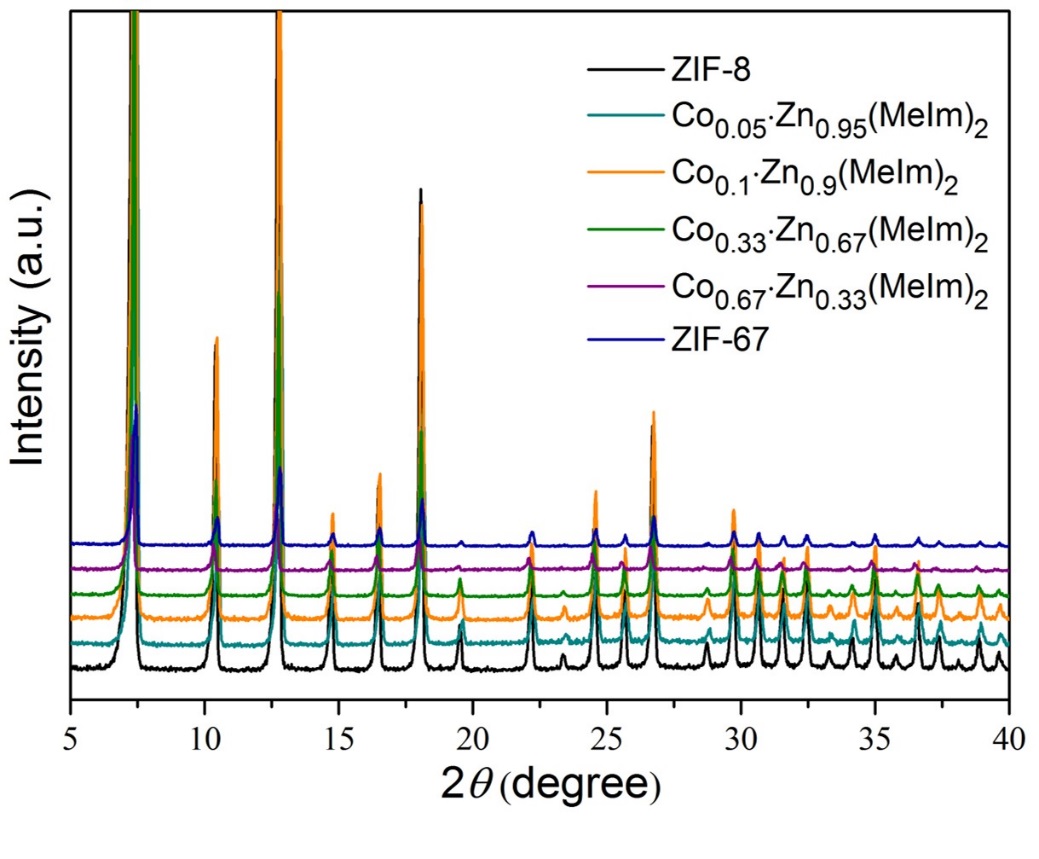


**Figure S2**. Wide-angle powder XRD patterns of the as-prepared ZIF-8, ZIF-67, and bimetallic ZIF (Co*_x_*·Zn_1-_*_x_*(MeIm)_2_) crystals.

**Figure S3**

**
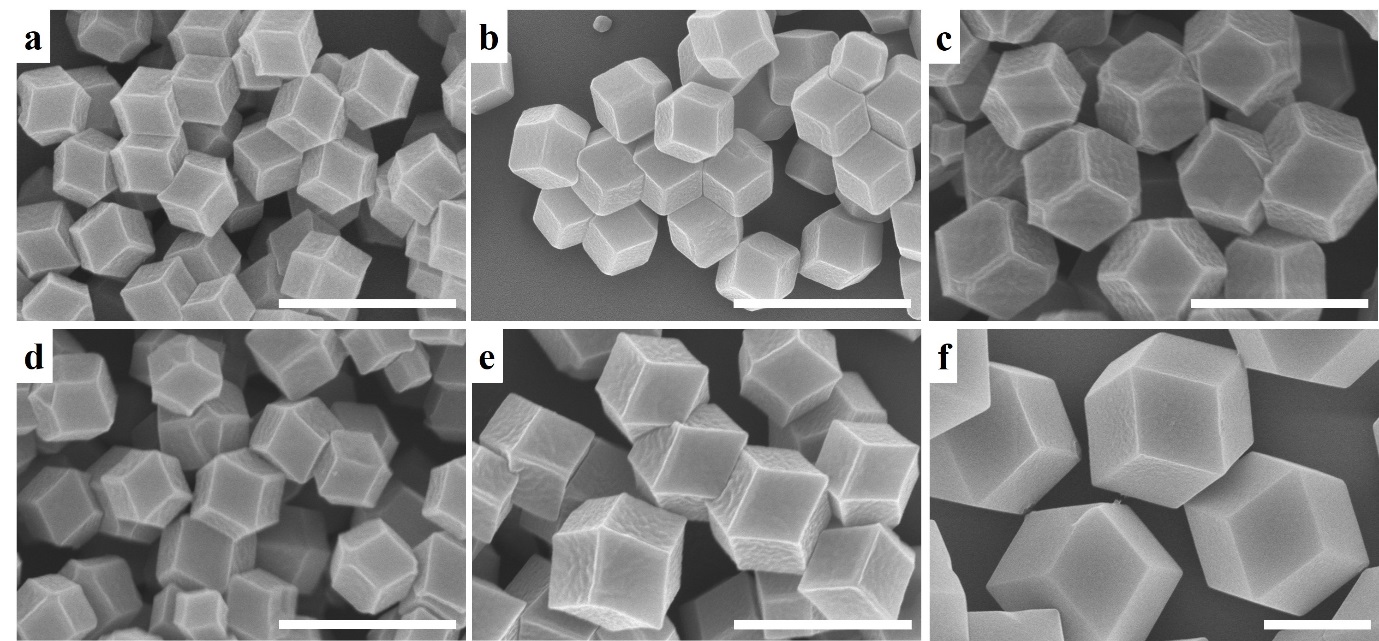
**

**Figure S3.** SEM images of the as-synthesized (a) ZIF-8, (b) Co_0.05_·Zn_0.95_(MeIm)_2_, (c) Co_0.1_·Zn_0.9_(MeIm)_2_, (d) Co_0.33_·Zn_0.67_(MeIm)_2_, (e) Co_0.67_·Zn_0.33_(MeIm)_2_, and (f) ZIF-67. The scale bars are all 1 μm.

**Figure S4**

**
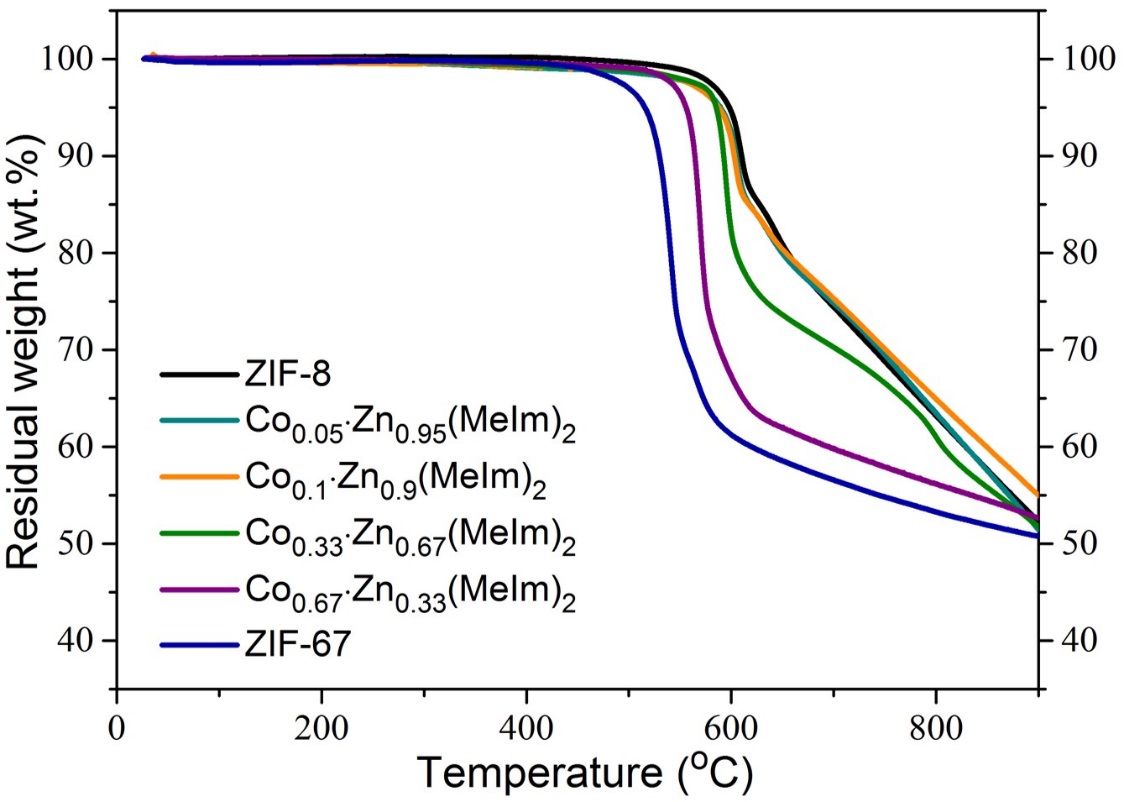
**

**Figure S4.** TG curves of ZIF-8, ZIF-67, and bimetallic ZIF (Co*_x_*·Zn_1-_*_x_*(MeIm)_2_) crystals measured under N_2_ atmospheres at a heating rate of 5 °C·min^-1^.

**Figure S5**

**
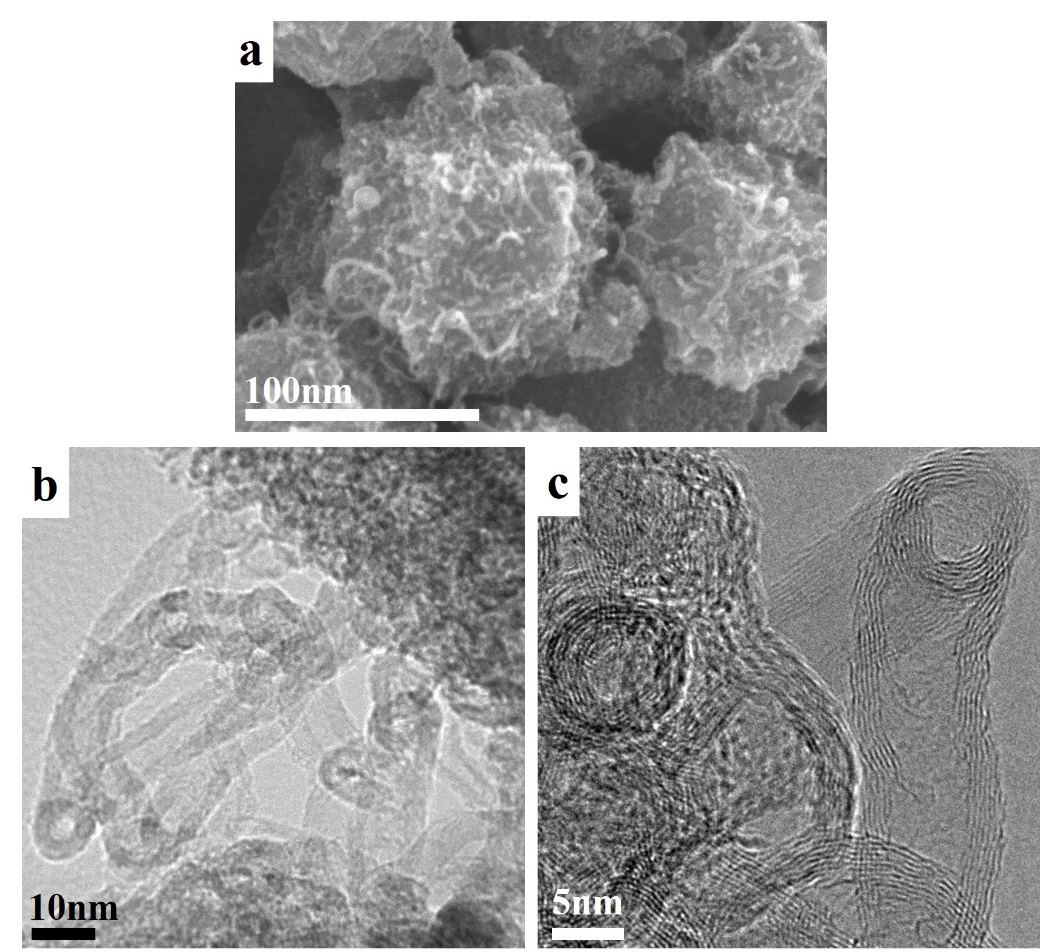
**

**Figure S5.** (a) SEM image and (b, c) high-resolution TEM images of sample C-2/1.

**Figure S6**

**
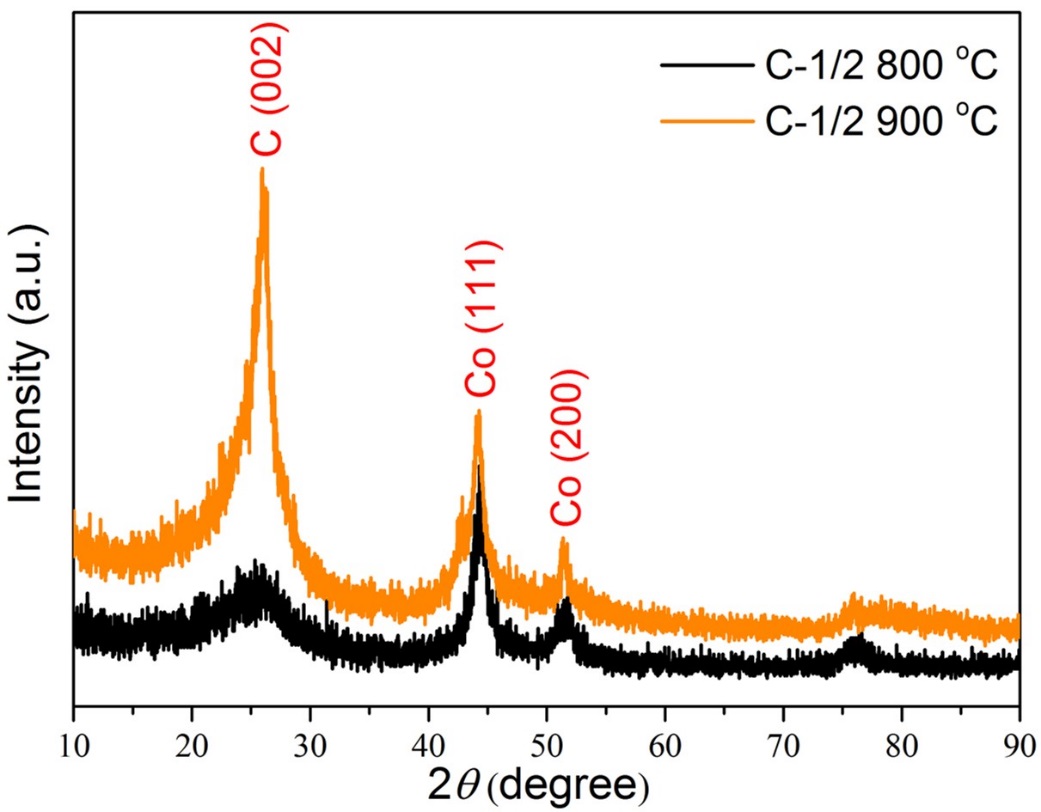
**

**Figure S6.** Wide-angle powder XRD patterns of the bimetallic-ZIF of Co_0.33_·Zn_0.67_(MeIm)_2_ derived carbon carbonized at 800 and 900 ºC, respectively.

**Figure S7**


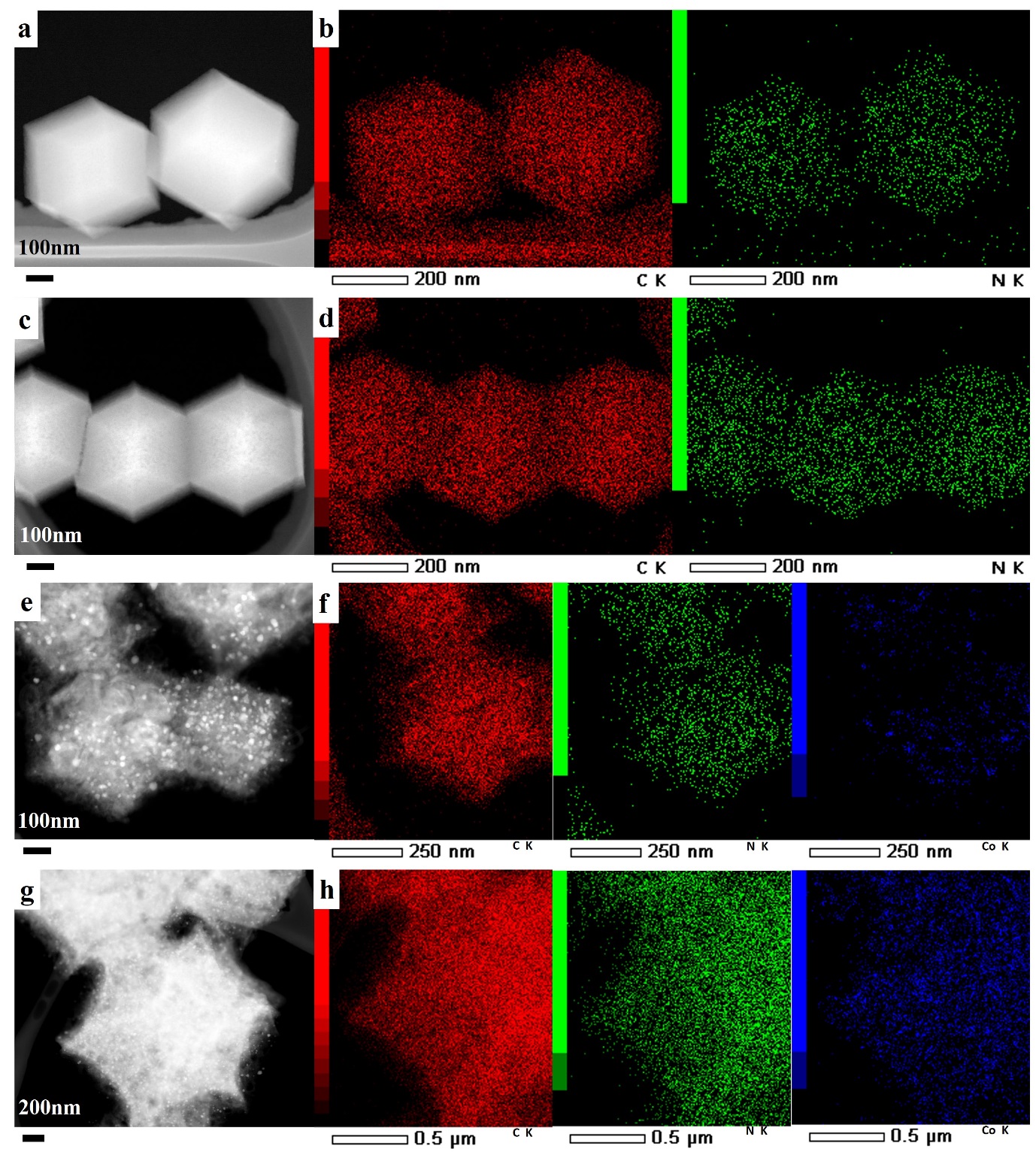


**Figure S7.** (a, c, e, g) HAADF STEM and (b, d, f, h) elemental mapping images of (a, b) C-ZIF-8, (c, d) C-1/9, (e, f) C-2/1, and (g, h) C-ZIF-67.

**Figure S8**


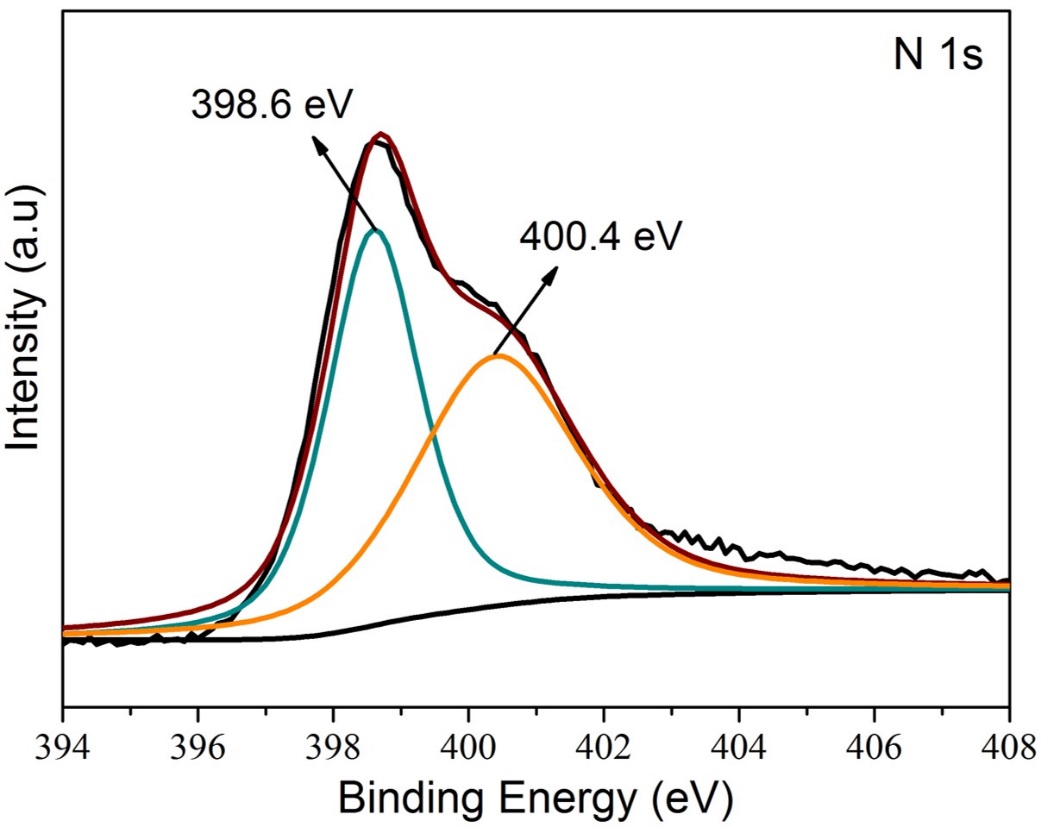


**Figure S8.** High-resolution XPS spectrum centered on the N 1s peak of the representative C-1/19 sample.

**Figure S9**

**
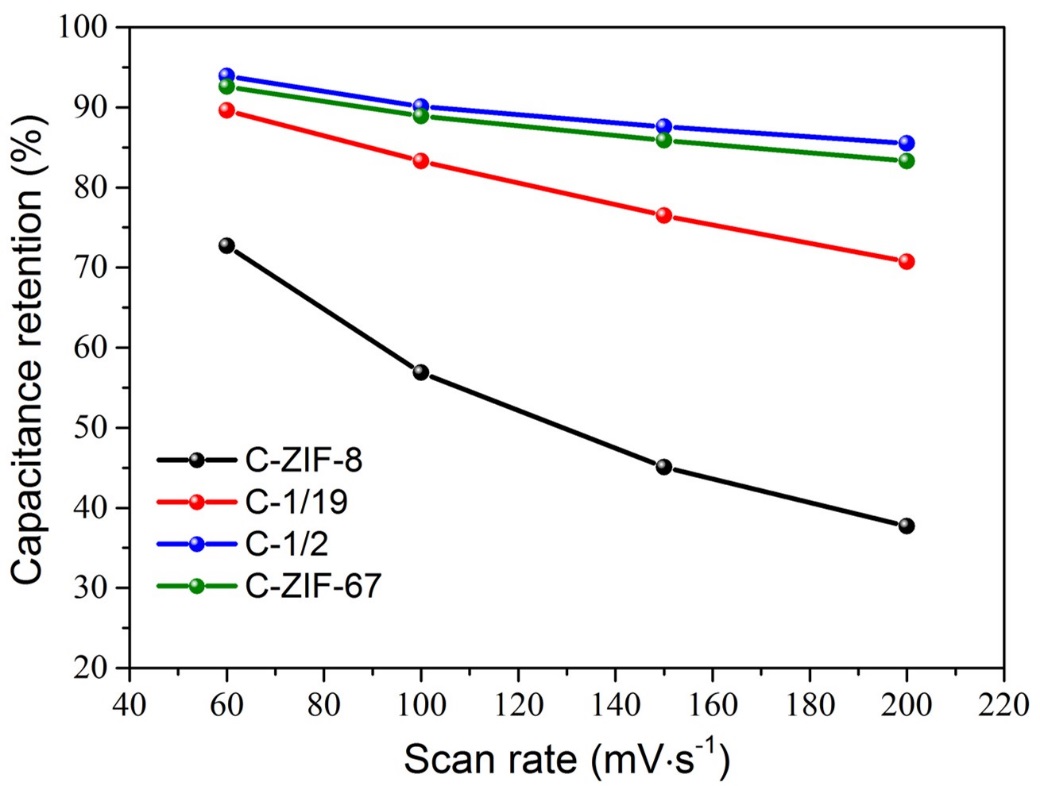
**

**Figure S9.** Volumetric capacitance retention of C-ZIF-8, C-1/19, C-1/2 and C-ZIF-67 samples as a function of the applied scan rates. The capacitance retention is the volumetric capacitance calculated at a higher scan rate compared to the initial scan rate of 20 mV∙s^-1^.

**Figure S10**

**
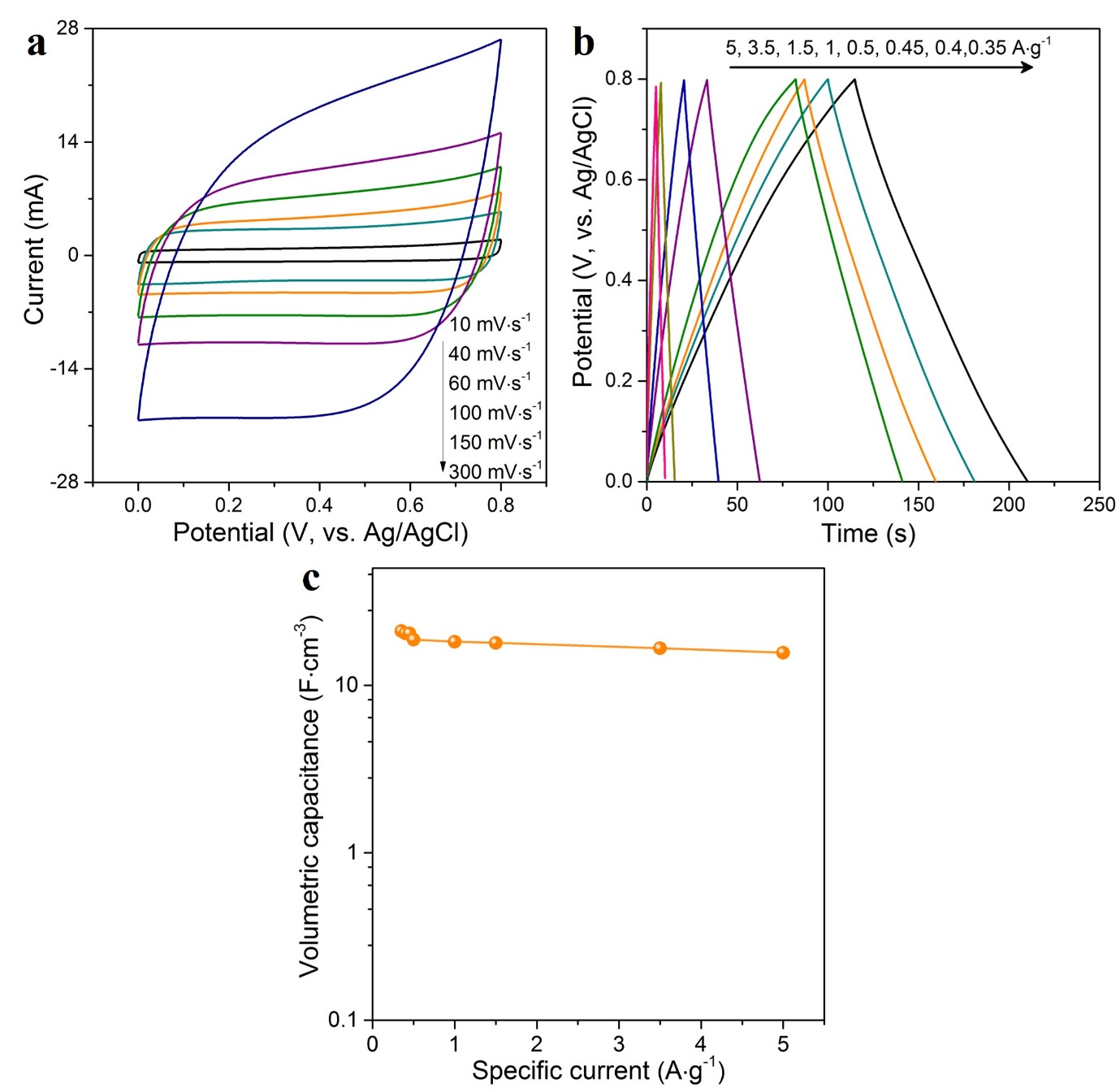
**

**Figure S10.** (a) CV curves, at different scan rates, of the symmetric supercapacitor cell (SSC) with nanoporous carbon (C-1/19 sample) positive and negative electrodes. The device was cycled within a potential window ranging from 0.0 to 0.8 V. (b) Galvanostatic charge-discharge curves with current density for the SSC. (c) Volumetric capacitance of the SSC as a function of the applied current densities.

**Notes for Figure S10:**

Among all of the samples, C-1/19 shows the high volumetric capacitance (95 F∙cm^-3^) with good capacitance retention (71%), further supercapacitor studies were carried out using this sample. A symmetric supercapacitor cell (SSC) was fabricated using C-1/19 for the positive and negative electrodes. **Figure S10a** shows the CV curves for the SSC at various scan rates ranging from 10 to 300 mV∙s^-1^. The CV shape is unaltered, even at high scan rates. This shows high stability and good capacitance retention for capacitor materials. The galvanostatic charge-discharge analysis were carried out at various applied current densities (**Figure S10b**). More interestingly, the charge-discharge curves show no electrode-potential drop (*IR* drop), even when the applied current density is increased up to 5 A∙g^-1^ (~15 times the initial current density), indicating the low internal ion-transport resistance^R1^. The capacitance values are found to be 21.1, 20.4, 20.3, 18.7, 18.2, 17.9, 17.9, and 15.6 F∙cm^-3^ at current densities of 0.35, 0.4, 0.45, 0.5, 1.0, 1.5, 3.5, and 5 A∙g^-1^, respectively (**Figure S10c**). C-1/19 shows a good volumetric capacitance of 21 F∙cm^-3^ at a current density of 0.35 A∙g^-1^, and a high capacitance retention of 76.5% at a current density as high as 5 A∙g^-1^. This clearly reveals that our carbon material can be used in high-rate operating devices with high volumetric capacitance and capacitance retention.

R1. Wang, D.-W., Li, F., Liu, M., Lu, G. Q. & Cheng, H.-M. 3D aperiodic hierarchical porous graphitic carbon material for high-rate electrochemical capacitive energy storage. *Angew. Chem., Int. Ed.* **47**, 373–376 (2008).
